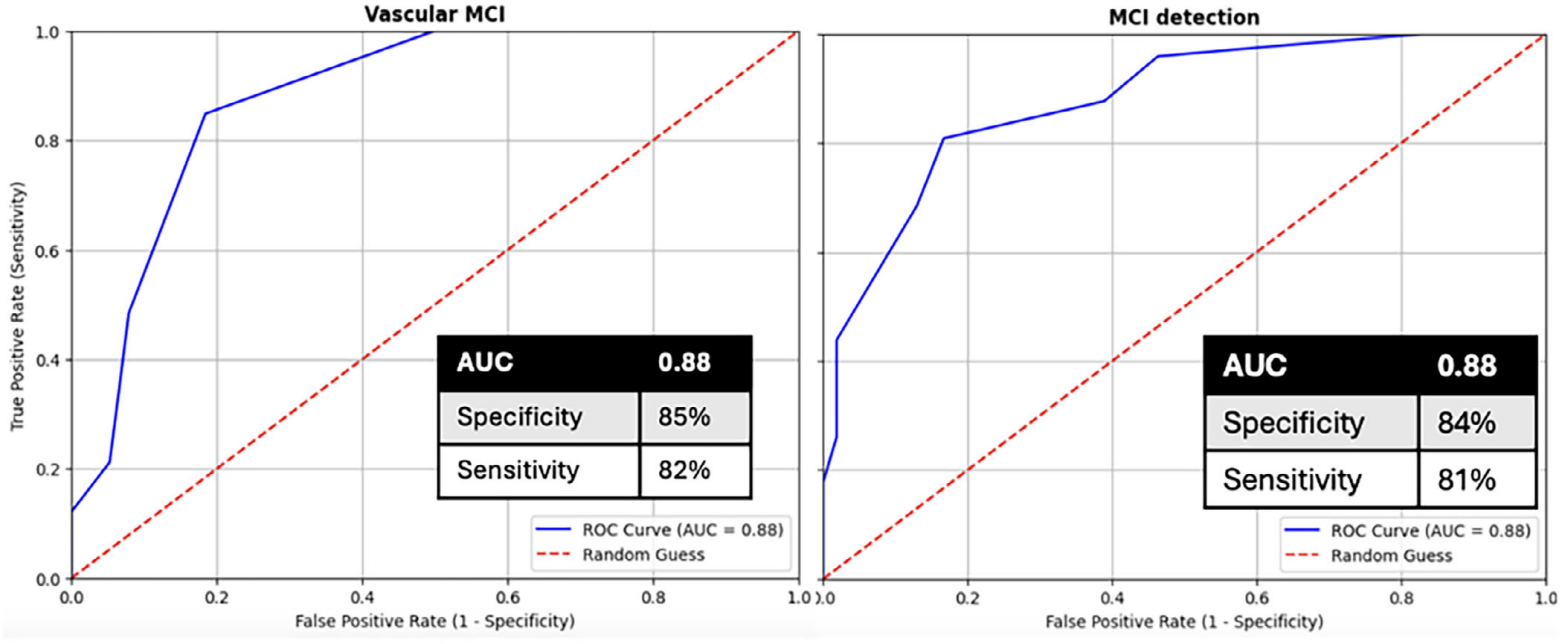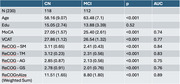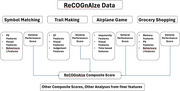# A novel digital assessment to detect Vascular, Alzheimer's and Mixed pathologies in Mild Cognitive Impairment

**DOI:** 10.1002/alz70857_107780

**Published:** 2025-12-26

**Authors:** Mohammed Adnan Azam, Ashwati Vipin, Yi Jin Leow, Gurveen Kaur Sandhu, Pricilia Tanoto, Nagaendran Kandiah

**Affiliations:** ^1^ Lee Kong Chian School of Medicine, Nanyang Technological University, Singapore, Singapore, Singapore; ^2^ Lee Kong Chian School of Medicine, Nanyang Technological University, Singapore, Singapore; ^3^ nil, nil, nil, Nicaragua; ^4^ Dementia Research Centre (Singapore), Lee Kong Chian School of Medicine, Nanyang Technological University, Singapore, Singapore; ^5^ Neuroscience and Mental Health Programme, Lee Kong Chian School of Medicine, Nanyang Technological University, Singapore, Singapore; ^6^ Duke‐NUS Medical School, National University of Singapore, Singapore, Singapore

## Abstract

**Background:**

Vascular Cognitive Impairment (VCI) is the second most common cause of dementia after Alzheimer's Disease (AD) (Gorelick et al.,2011). The coexistence of cerebrovascular pathology with AD pathology adds complexity to the diagnosis and treatment of VCI, underscoring the importance of early identification of MCI and its types ‐ Vascular, Alzheimer's and Mixed.

**Method:**

The data of 389 subjects from the Biomarker and Cognition Study (BIOCIS) at the Dementia Research Centre (Singapore), classified into Cognitively Normal (CN) and MCI, with detailed neuropsychological information and cognitive and behavioural domain impairment information was analysed. T2‐weighted FLAIR imaging was used to quantify subjects’ White Matter Hyperintensities (WMH) load. Plasma amyloid beta 42/40 ratio was used to quantify amyloid levels.

Subjects with MCI and high WMH load had significantly more executive dysfunction and lower processing speed along with higher impulse dyscontrol and increased apathy.

These domains were used to create 4 simplified multi‐domain digital games to assess different neuropsychological domains. Machine learning models were created and trained on data to differentiate the different pathologies based on neuropsychological features derived from the cognitive games.

230 new subjects (112 with MCI) were recruited for the validation of the developed digital games. Out of the 112 subjects with MCI, 56 had a high WMH load and they were in the Vascular MCI group. The Vascular MCI group was then split into Mixed (*n* = 26, high Amyloid) and Vascular (*n* = 26, low Amyloid).

**Result:**

A combined game score derived from the game features, was able to detect MCI from CN with an Area Under the Curve (AUC) of 0.89 as well as detect Vascular from non‐Vascular with an AUC of 0.88. Within the Vascular group, it was able to detect Mixed MCI with an AUC of 0.9 and within the non‐Vascular group it was able to detect Alzheimer's MCI (high Amyloid) with an AUC of 0.9.

**Conclusion:**

The ReCOGnAIze app has shown promising results in detecting MCI and differentiating Vascular, Alzheimer's and Mixed which can guide decision making for further evaluations for clinical diagnoses and interventions.